# COVID-19 Cerebral Vasculitis With Extensive Involvement of Anterior Circulation and Sparing of Posterior Circulation

**DOI:** 10.7759/cureus.86818

**Published:** 2025-06-26

**Authors:** Thomas E Kent, Sohil H Patel, Prem P Batchala, Joseph H Donahue, Thomas J Eluvathingal Muttikkal

**Affiliations:** 1 School of Medicine, University of Florida, Gainesville, USA; 2 Department of Radiology and Medical Imaging, University of Virginia Health System, Charlottesville, USA

**Keywords:** acute ischemic stroke, covid-19, posterior circulation, vasculitis, vessel wall imaging

## Abstract

COVID-19 infection is associated with stroke due to various proposed mechanisms, including cerebral vasculitis, arterial and venous thrombosis. Vessel wall imaging, although not pathognomonic, interpreted in the appropriate clinical context and supportive laboratory findings, can help clarify the diagnosis and differentiate the etiology from other mechanisms of CNS involvement. We describe a patient with extensive anterior circulation vasculitis and ischemic stroke in the setting of COVID-19, with sparing of the posterior circulation.

## Introduction

COVID-19-associated neurological and vascular complications include acute ischemic stroke (AIS) due to arterial thrombosis, cerebral venous thrombosis, hemodynamic hypoperfusion-hypoxic injury, vasculitis, acute disseminated encephalomyelitis, encephalitis, and microhemorrhages [1]. Vascular narrowing on angiography and concentric enhancement of large and medium intracranial vessels on MRI are reported as evidence for COVID-19-associated vasculopathy and vasculitis, respectively [1-4]. The pattern of vessel wall enhancement often helps to differentiate the etiologies. Vessel wall enhancement, including a circumferential pattern, is not pathognomonic of vasculitis and has to be interpreted in the appropriate clinical background and supportive laboratory evaluations [5-9].

## Case presentation

**Figure 1:**
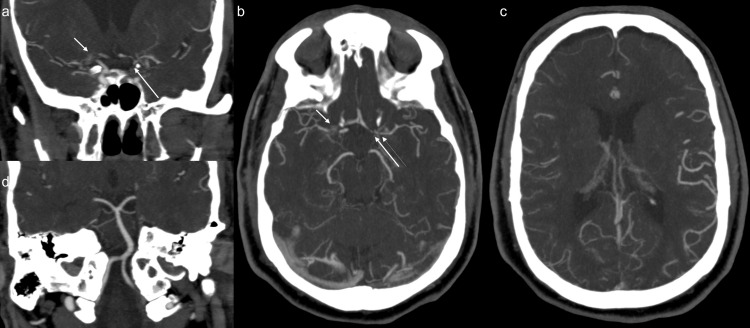
CTA head maximum intensity projection (MIP) shows occlusion and multivessel stenosis of the anterior circulation, with widely patent posterior circulation. (a) CTA coronal MIP shows occlusion of right internal carotid artery (ICA) terminus and origins of right anterior cerebral artery (ACA) and middle cerebral artery (MCA) (short arrow), and severe stenosis of supraclinoid portion of the left ICA (long arrow). (b) CTA axial MIP shows occlusion of right ICA terminus and origins of right ACA and MCA (short arrow), and severe stenosis of A1 segment of left ACA (long thick arrow), severe narrowing of the origin (long thin arrow) of left MCA. In addition, there is severe stenosis of proximal M2 branches on the right and moderate narrowing of the distal M1 and proximal M2 branches on the left. (c) CTA axial MIP shows diffuse narrowing and poor opacification of M3 and distal branches of right MCA. (d) CTA coronal MIP shows widely patent posterior circulation.

**Figure 2:**
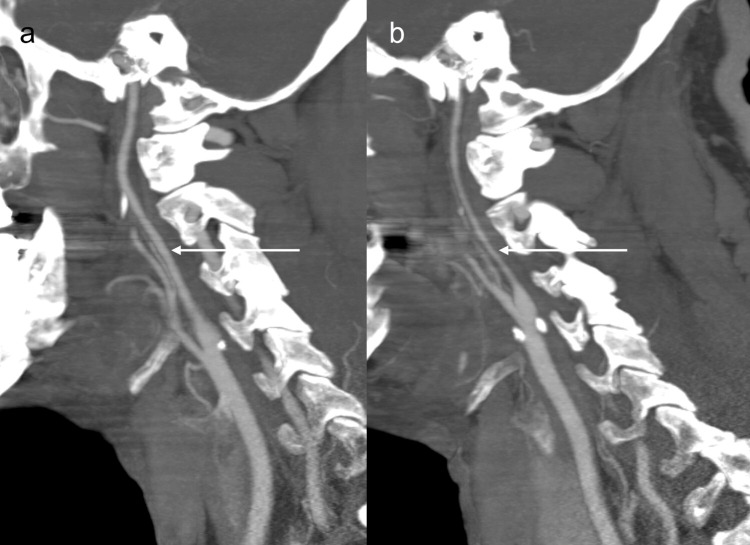
CTA sagittal maximum intensity projection (MIP) shows cervical internal carotid arteries (ICAs). (a) CTA sagittal MIP shows right cervical ICA without stenosis (arrow). (b) CTA sagittal MIP shows diffuse narrowing of left cervical ICA (arrow).

A 64-year-old male presented to the emergency department at a regional hospital with shortness of breath. The patient was diagnosed with COVID at his routine workplace screening 10 days prior. He subsequently developed symptoms and was having shortness of breath and chest pain, which got worse, and also had a nonproductive cough and fever, and had been taking ibuprofen. He had received one dose of COVID vaccination nine months prior. On presentation, his pulse oxygen saturation was 74% on room air, blood pressure 161/121, and he had a respiratory rate of 21. Chest radiograph showed bilateral pulmonary infiltrates. The patient was admitted for acute respiratory failure with hypoxemia. He was placed on high-flow oxygen at 30 L/minute, started on intravenous dexamethasone, intravenous remdesivir, and scheduled fluticasone/salmeterol inhaler. Over a period of 12 hours, his respiratory status declined, oxygen requirement went up to 70 L/minute, and he was subsequently placed on bilevel positive airway pressure (BiPAP). The next morning, he was noted to have left-sided weakness. The stroke code alert was initiated. On tele-stroke evaluation, the patient had National Institutes of Health Stroke Scale (NIHSS) = 7 (one each for motor left arm and left leg, one for inattention/extinction, one for language, one for facial palsy, one for level of consciousness (LOC) commands, one for LOC questions). CT head and CT angiography (CTA) of the head and neck were obtained. CT head did not show any acute abnormality, with an Alberta Stroke Program Early CT (ASPECT) score of 10. CTA of the head and neck (Figure 1) showed occlusion of the right internal carotid artery (ICA) terminus, severe stenosis of the supraclinoid portion of the left ICA, multifocal severe stenosis of the bilateral anterior and middle cerebral arteries, and diffuse stenosis of the cervical portion of the left ICA (Figure 2).

**Figure 3:**
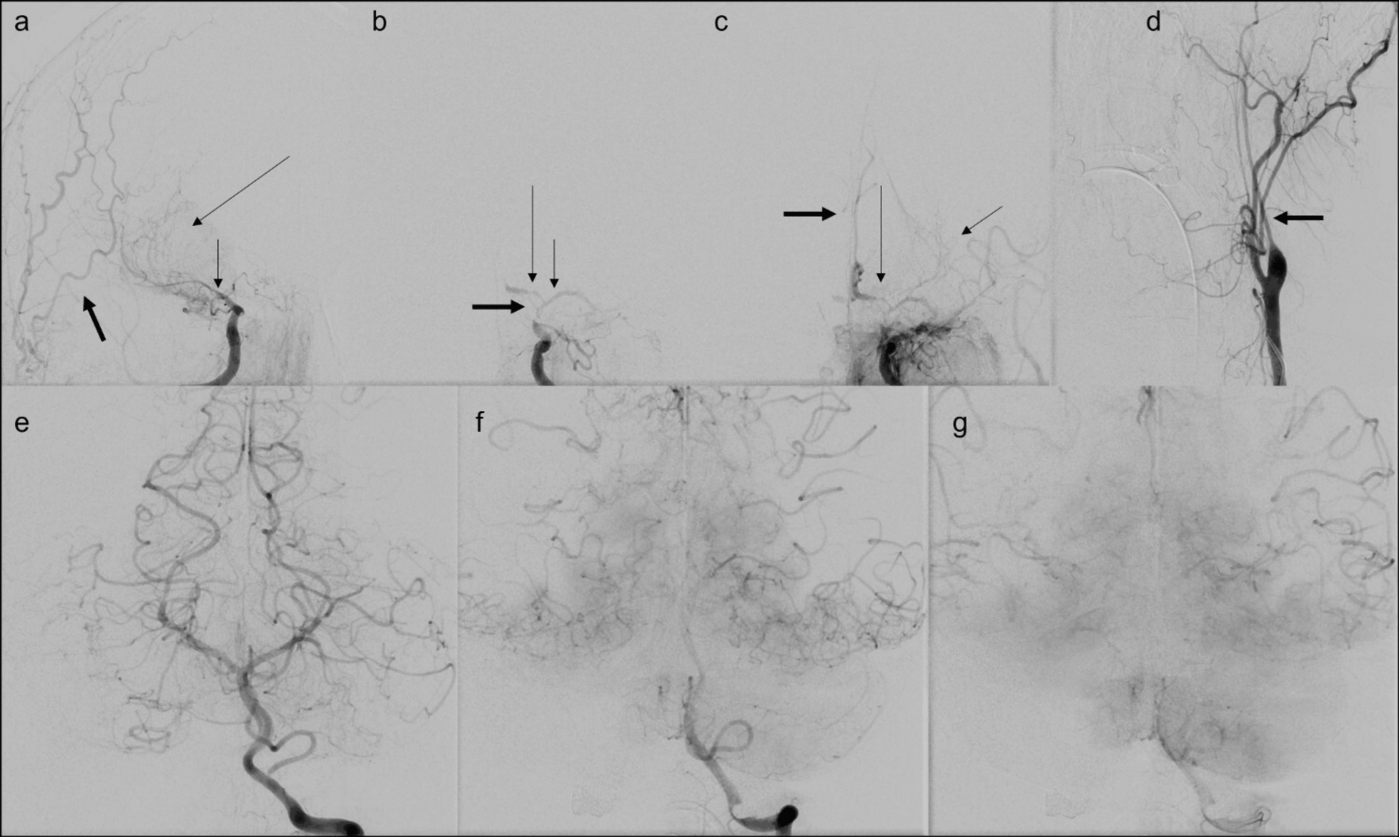
Catheter angiography shows multivessel stenosis in anterior circulation with no stenosis in posterior circulation. (a) Catheter angiography anterior oblique projection shows severe stenosis of the right ICA terminus as well as the right middle cerebral artery (MCA) M1 segment (short thin arrow) with delayed faint partial filling of the right MCA (long thin arrow) and no evidence of filling of the right anterior cerebral artery (ACA). External carotid artery (ECA) branches are seen as opacified, denser, and earlier than MCA branches (thick arrow). (b) Catheter angiography anterior projection shows severe stenosis of the communicating segment and terminus of left internal carotid artery (ICA) (thick arrow), severe stenosis of the M1 segment of left MCA (short thin arrow), and A1 segment of left ACA (long thin arrow). (c) Catheter angiography anterior projection shows delayed faint partial filling of the left MCA (short thin arrow), severe stenosis of the left A1 ACA segment (long thin arrow) with delayed filling of the ACA distally and faint delayed filling of the contralateral ACA (thick arrow) through anterior communicating artery. (d) Catheter angiography anterior projection shows diffuse stenosis of cervical segment of left ICA. (e, f, g) Catheter angiography anterior projection shows cortical anastomotic filling of bilateral temporal, parietal, and posterior frontal territories of MCAs as well as retrograde filling of the ACAs from left vertebral artery injection, with no evidence of stenosis in posterior circulation.

Posterior circulation was normal (Figure 1d). The patient was transferred to our hospital, a comprehensive stroke center, for further management. The patient was transferred intubated and sedated, limiting the neurologic examination. Neurological examination revealed PERRL (Pupils Equal, Round, Reactive to Light). Extraocular muscles are intact to the vestibulo-ocular reflex bilaterally, not rousable to voice, light, physical stimulation, or noxious stimulation. There was no withdrawal to noxious stimuli in all four extremities. There was no ankle clonus. Muscle stretch reflexes of biceps, brachioradialis, triceps, patellar, and Achilles were 2+. Plantar reflex was downgoing bilaterally. Differential considerations for the vascular findings included diffuse intracranial arteriopathy, including reversible cerebral vasoconstriction syndrome (RCVS), given the hypertension and prior case reports of occurrence of RCVS in COVID, primary angiitis of central nervous system (PACNS), secondary CNS vasculitis due to systemic inflammatory or infectious process including COVID-19, and severe atherosclerotic disease. Patient underwent catheter angiography for further characterization, which also demonstrated multifocal stenosis in the anterior circulation (Figure 3), without any response to verapamil injection.

There was no evidence of typical atherosclerotic disease in the neck on CTA. The pattern of narrowing on catheter angiography was not that of atherosclerotic disease. CTA of the abdomen and pelvis was done the next day, to look for additional areas of vascular involvement, which showed long-segment non-atherosclerotic narrowing of the common hepatic artery with surrounding fat stranding, concerning for vasculitis, which argued against the diagnosis of PACNS. CT pulmonary angiography was done to rule out pulmonary embolus, showed bilateral consolidations and diffuse geographic ground glass opacities consistent with COVID infection. Remdesivir was discontinued as the patient was more than 10 days out of his initial symptoms. IV dexamethasone was continued for 10 days. Tocilizumab was also administered. MRI of the brain was done the next day when the patient was stable enough to undergo MRI.

**Figure 4:**
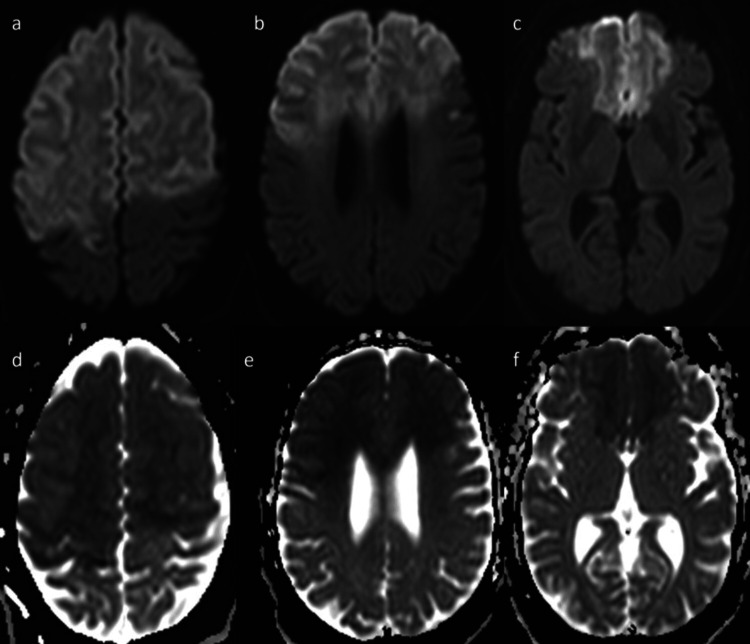
Brain MRI shows confluent early subacute infarcts involving bilateral frontal lobes and right parietal lobe with relative sparing of the inferolateral aspect, and involvement of caudate nuclei. (a) Brain MRI axial diffusion-weighted imaging (DWI) shows confluent hyperintense signal with mild swelling involving bilateral frontal lobes and anterior portion of right parietal lobe. (b) Brain MRI axial DWI shows confluent hyperintense signal with mild swelling involving bilateral frontal lobes, with relative sparing of the inferolateral aspect. (c) Brain MRI axial DWI shows confluent hyperintense signal with mild swelling involving bilateral frontal lobes with relative sparing of the inferolateral aspect, and involvement of caudate nuclei. (d, e, f) MRI brain ADC shows corresponding low signal intensity.

MRI showed confluent anterior circulation territory early subacute infarcts involving bilateral frontal lobes, right parietal lobe, and caudate nuclei (Figure 4). 

**Figure 5:**
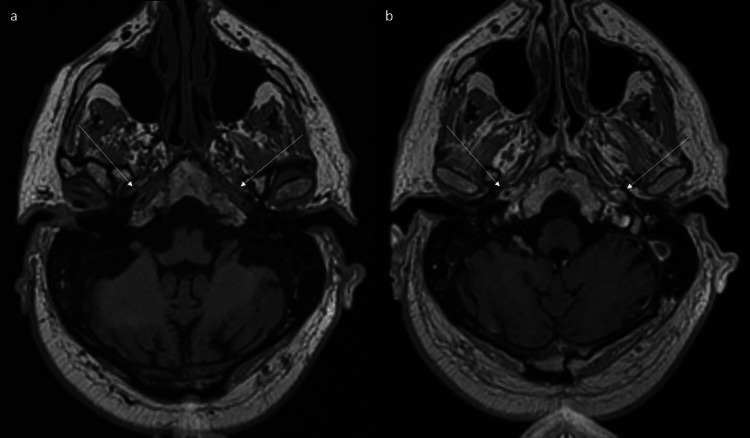
MRI shows abnormal wall thickening and enhancement of petrous portion of internal carotid arteries. (a) Axial non-contrast and (b) contrast-enhanced T1 space sequences show abnormal wall thickening and enhancement of petrous portion of internal carotid arteries (arrows).

**Figure 6:**
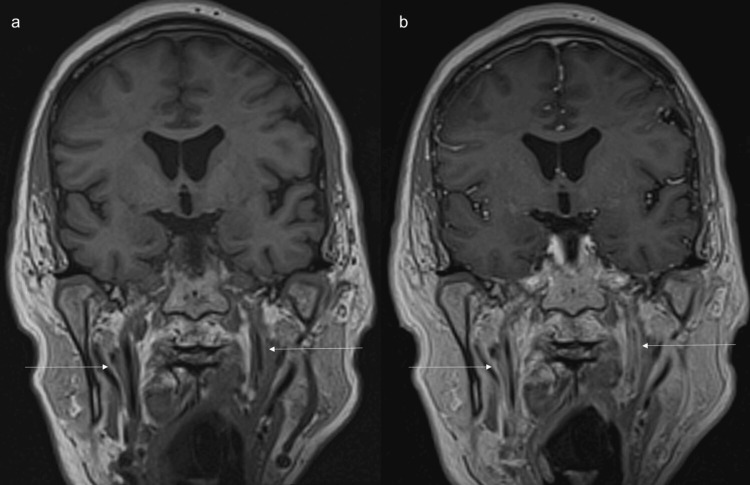
MRI shows long-segment abnormal wall thickening and enhancement of cervical portion of internal carotid arteries. (a) Coronal non-contrast and (b) contrast enhanced T1 space sequences show long segment abnormal wall thickening and enhancement of cervical portion of internal carotid arteries(arrows).

Contrast-enhanced T1 space sequence (Figure 5 and Figure 6) showed abnormal wall thickening and enhancement of intracranial and cervical portions of internal carotid arteries, findings compatible with vasculitis in the clinical context and laboratory evaluation (Table 1). The lack of response to verapamil on catheter angiography, along with the extensive wall thickening and enhancement of cervical and intracranial portions of ICA, and involvement of the common hepatic artery, made the possibility of RCVS quite unlikely.

**Figure 7:**
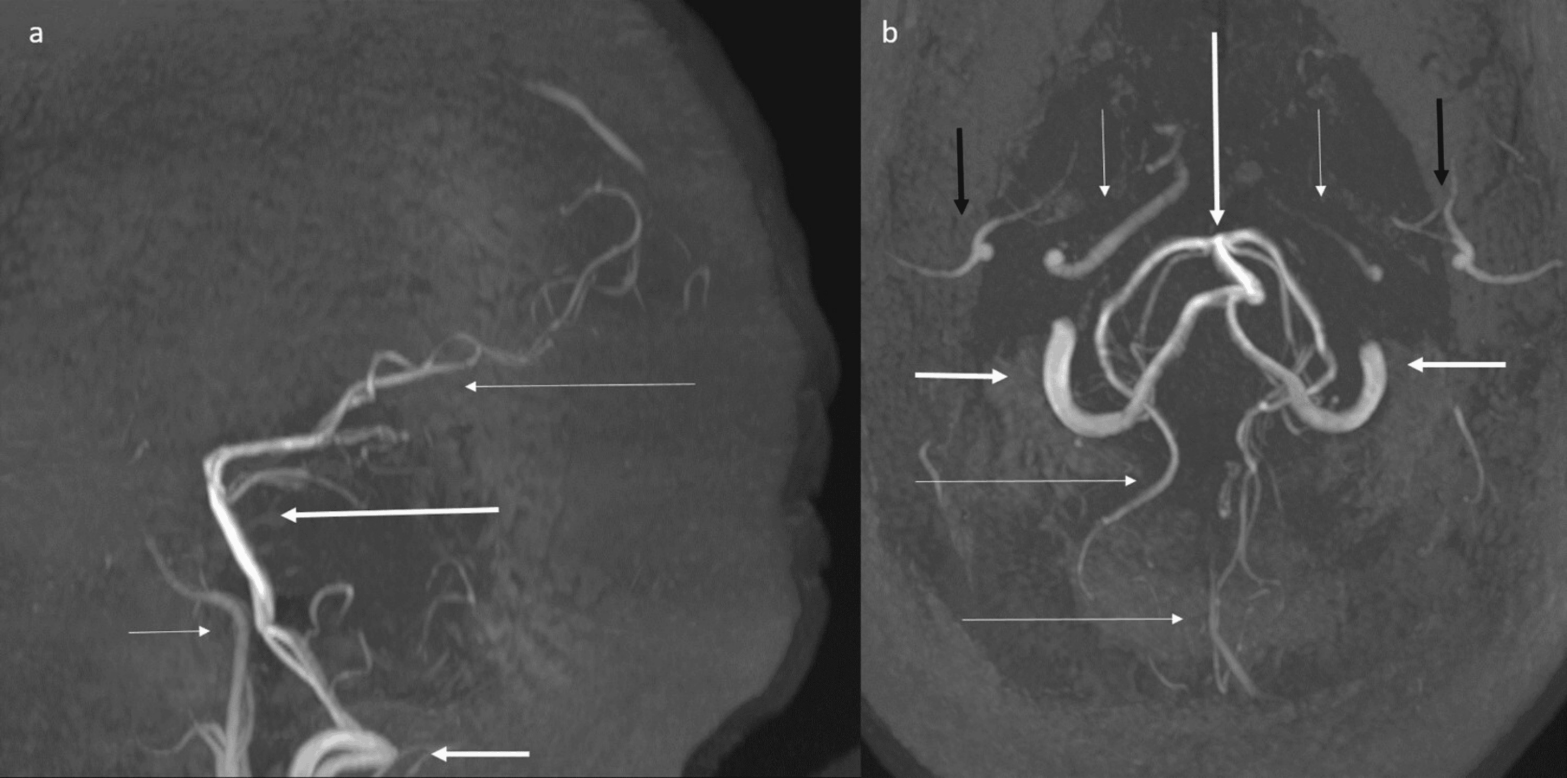
Time of flight (TOF) magnetic resonance angiography (MRA) maximum intensity projection (MIP) shows absence of flow-related enhancement of anterior and middle cerebral arteries, with attenuated appearance of internal carotid arteries, and prominent posterior circulation arteries. (a) Time-of-flight (TOF) MRA sagittal MIP shows absence of flow related enhancement of anterior and middle cerebral arteries, with attenuated appearance of internal carotid arteries (short thin arrow), and prominent vertebral (short thick arrow), basilar (long thick arrow) and posterior cerebral arteries (long thin arrow). (b) TOF MRA axial MIP shows absence of flow-related enhancement of anterior and middle cerebral arteries, with attenuated appearance of internal carotid arteries (short thin arrows), and prominent vertebral (short thick arrows), basilar (long thick arrows), and posterior cerebral arteries (long thin arrows). ECA branches are denoted by black arrows.

Time of flight (TOF) magnetic resonance angiography (MRA) (Figure 7) showed no flow-related enhancement of anterior and middle cerebral arteries with attenuated appearance of internal carotid arteries, and prominent posterior circulation arteries providing collateral supply.

Serologic and CSF evaluation was unrevealing for a specific etiology of vasculitis (Table 1).

**Table 1:** Serologic and CSF evaluation Ab, antibody; AI, antibody index; HBcAb, hepatitis B core antibody; HBsAb, hepatitis B surface antibody; HBsAg, hepatitis B surface antigen; LDH, lactate dehydrogenase; PCR, polymerase chain reaction; RNP, ribonucleoprotein; RSV, respiratory syncytial virus

Tests with reference range and units	Result
Antinuclear antibodies	Negative
Extractable nuclear antigen (ENA) profile	Negative
Smith Ab <1.0 AI	<0.2
RNP Ab <1.0 AI	<0.2
SSA (Ro) Ab <1.0 AI	<0.2
SSB (La) Ab <1.0 AI	<0.2
Scl 70 Ab <1.0 AI	<0.2
Jo 1 Ab <1.0 AI	<0.2
Antichromatin IgG Ab <1.0 AI	<0.2
Ribosomal P Ab <1.0 AI	<0.2
Centromere Ab Screen <1.0 AI	<0.2
Anti-Smith Ag RNP <1.0 AI	<0.2
Anti-neutrophil cytoplasmic antibody	Negative
Myeloperoxidase <1.0 AI	<0.2
Proteinase 3 <1.0 AI	<0.2
HIV serology	Negative
HBsAg	Non-reactive
HBsAb	2.6 mIU/mL
HBcAb	Non-reactive
Hepatitis C Ab	Non-reactive
SARS-CoV-2 PCR	Positive
RSV PCR	Negative
Influenza A	Negative
Influenza B	Negative
Lyme serology	Negative
Syphilis total Ab <0.9 AI	<0.2
Blood culture	No growth
CRP <10.0 mg/L	116.9
ESR 0-30 mm/hour	39
Ferritin 17.9-464 ng/mL	1810
LDH 313-618 U/L	1051
Blood glucose 74-99 mg/dL	246
CSF	
Opening pressure	23
Color and appearance	Colorless, clear
WBC 0-5/μL	2
RBC 0-5/μL	0
Lymphocyte	52%
Monocyte/macrophage	48%
Protein 15-25 mg/dL	34.0
Glucose 40-70 mg/dL	157.0
Lactate 0.5-2.2 mmol/L	2.5
IgG index ≤0.85	0.55
IgG ≤8.1 mg/dL	2.0

The patient was started on 1 g IV Solu-Medrol for vasculitis, which was given for four days, followed by a prolonged taper of prednisolone. This was done despite relatively bland CSF findings, given the imaging findings, laboratory evaluation, and prior case reports of COVID-19 CNS vasculitis with bland CSF that did not suggest CNS inflammation but were later confirmed as vasculitis and successfully treated with high-dose IV steroids. The patient was also started on aspirin. Follow-up CT head was done two days after the MRI showed extensive bilateral anterior circulation infarct without any significant change in the extent or distribution, and no evidence of hemorrhagic transformation. The patient continued to be comatose three days off sedation, not rousable to voice, light physical stimulation, or noxious stimulation. Pupils were round and reacting to light, with intact corneal reflex bilaterally. There was flaccid tone in the upper extremities and diminished tone in the lower extremities, with no spontaneous or purposeful movement, no tremors, fasciculations, or myoclonus. There was no response to noxious stimuli in all four extremities. There was no clonus. Bilateral upgoing plantars were present, and there was triple flexion bilaterally. At the time of discharge, after about three months of hospital stay, the patient was alert, had flattened affect, and was quadriplegic. The patient showed little improvement in neurologic function throughout admission due to the extensive infarcts and was discharged home on home health services, with tracheostomy and percutaneous endoscopic gastrostomy tube.

## Discussion

Viral infection can precipitate cerebral vasculitis, which can then lead to AIS. In several cases, in patients with COVID-19 with AIS, there have been findings of intracranial vessel wall thickening and intracranial vascular stenosis [1-4]. Patchy/punctate ischemic changes in brain imaging in the topographic pattern of vasculitis are not sufficient for the diagnosis of vasculitis [2]. Vessel wall narrowing as demonstrated by catheter angiography, CTA, or MR angiography is sufficient for the diagnosis of vasculopathy, although not necessarily vasculitis, while concentric vessel wall enhancement is suggestive of vasculitis [2]. The pattern and spatial distribution of vessel wall enhancement help to differentiate between different etiologies [5-8]. Atherosclerotic plaques usually show eccentric heterogenous focal enhancement compared to concentric wall thickening and enhancement in vasculitis. RCVS mostly shows concentric wall thickening without or with mild contrast enhancement compared to intense enhancement in vasculitis. However, atherosclerotic plaques can show concentric enhancement, and concentric intense thin-wall enhancement is known to occur in intracranial vessels in RCVS [5-9]. Thus, vessel wall enhancement, even the circumferential pattern, although suggestive, is not pathognomonic of vasculitis and has to be interpreted with the clinical picture and laboratory evaluations. In our case, the patient had extensive diffuse enhancement of the cervical internal carotid arteries bilaterally with severe wall thickening, in addition to the involvement of anterior circulation arteries intracranially. Such profound wall thickening and enhancement involving the entirety of bilateral cervical arteries differentiates it from RCVS, which uncommonly affects the cervical internal carotid arteries, and from atherosclerotic disease, especially in the presence of severe COVID and elevated inflammatory markers such as Ferritin, LDH, and CRP, pointing to cytokine storm [10]. In addition, there was non-atheromatous narrowing of the common hepatic artery with adjacent fat stranding, essentially ruling out PACNS. The imaging findings in the clinical context, along with supportive laboratory evidence, were compatible with vasculitis.

Theories for COVID-19-associated vasculitis include indirect effects through systemic immune dysregulation, such as cytokine storm, and associated coagulopathy, or direct invasion of blood vessels by the infectious agent binding to proteins, such as the angiotensin-converting-enzyme 2 (ACE2) receptor, expressed on the cerebrovascular endothelial cells [2,11,12].

In our case, the reason for the differential involvement between the anterior and posterior circulation is unknown. Such an extensive anterior circulation involvement juxtaposed with posterior non-involvement has not been described in the literature to the best of our knowledge and raises the possibility of differential affinity to anterior circulation vasculature, the mechanism of which remains unexplained. No studies to date have shown differential expression of ACE2 between the anterior and posterior circulation. In general, and in COVID-19-associated AIS, the anterior circulation is also more commonly affected [13]. In a large study, the prevalence of AIS in patients with COVID-19 (1.3%) was only slightly higher compared to patients without concurrent COVID-19 infection (1.0%), suggesting the significance of other predisposing, cardiovascular factors [14]. Regarding posterior circulation vasculitis involvement, a retrospective study found circumferential vessel wall enhancement in the posterior circulation, including the vertebral arteries, in 85% of patients with COVID-19 associated encephalopathy [15], without any involvement of anterior circulation, although there has been a question whether this represented true evidence of vasculitis or enhancement of vaso vasorum [16]. Table 2 shows the distribution of intracranial vessels involved [3,4,17-23].

**Table 2:** Intracranial vascular involvement - anterior versus posterior circulation ACA, anterior cerebral artery; Ant, anterior; b/l, bilateral; MCA, middle cerebral artery; PCA, posterior cerebral artery; Post, posterior

Publications	No. of patients with vessel wall enhancement	Vessels involved	Ant	Post	Ant and post
Mazzacane et al. [4]	3	Azygous ACA	2	-	1
		Right M1-M2			
		Anterior and posterior, b/l			
Appavu et al. [17]	1	Left ICA	1	-	-
Okrzeja et al. [18]	7	Not described	-	-	-
Dixon et al. [19]	1	ACAs, MCAs, vertebrals, basilar	-	-	1
Lersy et al. [3]	11	Left MCA, b/l PCA, basilar	-	-	1
		Left MCA	1	-	-
		Bilateral MCA, basilar	-	-	1
		Left MCA, basilar	-	-	1
		B/l MCA, basilar	-	-	1
		Basilar	-	1	-
		B/l MCA, b/l PCA, basilar	-	-	1
		B/l MCA, Basilar	-	-	1
		B/l MCA, b/l PCA	-	-	1
		Basilar	-	1	-
		B/l MCA, b/l PCA	-	-	1
Raban et al. [20]	1	Left MCA	1	-	-
Mirzaee et al. [21]	1	Left MCA	1	-	-
Keller et al. [22]	3	Left MCA and right PCA	1	-	1
		Right MCA			
		B/l, unclear whether ant or post			
Gulko et al. [23]	1	Left MCA	1	-	-

In an autopsy series with 36 cases, 32 had involvement of both anterior and posterior circulation, three cases had involvement of posterior circulation, and one case had involvement of anterior circulation [24].

Studies have demonstrated improvement of vessel wall enhancement following treatment [4,19,25,26]. The diagnosis of cerebral vasculitis is important because appropriate management can lead to reversal of vascular abnormalities, prevention of neurological complications, and clinical improvement [19,26,27]. In case reports and case series in patients with COVID with vessel wall enhancement suggestive of vasculitis, neurological outcome was variable and appears to be related to the extent and site of infarct (Table 3) [4,17,19-23].

**Table 3:** COVID vasculitis and neurological outcome ACA, anterior cerebral artery; b/l, bilateral; GCS, Glasgow Coma Scale; MCA, middle cerebral artery; mRS, Modified Rankin Scale; NIHSS, National Institutes of Health Stroke Scale; PCA, posterior cerebral artery

Publications	No. of patients with vessel wall enhancement	Vessel wall enhancement	MRI findings	Presentation	Outcome
Mazzacane et al. [4]	3	Azygous ACA	Small b/l ACA infarcts	NIHSS 3	mRS at three months - 1
		Right M1-M2	Right MCA acute infarct; subacute left frontal infarct	NIHSS 10	mRS at three months - 2
		Anterior and posterior, b/l	B/l small acute infarcts	NIHSS 1	mRS at three months - 1
Appavu et al. [17]	1	Left ICA	B/l small MCA infarcts	Mixed receptive-expressive aphasia and dysarthria, left hemiparesis	mRS - 4, with residual mixed receptive-expressive aphasia and dysarthria
Dixon et al. [19]	1	ACAs, MCAs, vertebrals, basilar	Subacute right MCA and b/l PCA infarcts	GCS 8 (E4, V2, M2)	Alert, appropriate speech, oriented to place and person, moving all limbs.
Raban et al. [20]	1	Left MCA	Small subacute left MCA infarcts	Mild encephalopathy, moderate right hemiparesis	Discharged after six days, encephalopathy resolved
Mirzaee et al. [21]	1	Left MCA	Left basal ganglia and internal capsule infarct	Right hemiparesis and dysarthria	Persistent hemiparesis
Keller et al. [22]	3	Left MCA and right PCA	Scattered subarachnoid hemorrhage	Delayed wake-up, myoclonic movements, later delirium	Memory and concentration problems
		B/l	B/l early subacute infarcts in different vascular territories, microbleeds	Right hemiparesis, dysarthria, delayed wake-up	Slight tetraparesis, temporarily delirious, slightly disoriented
		Right MCA	Small early subacute b/l MCA infarcts, microbleeds	Dysarthria, right facial palsy, right hemiparesis, delayed wake-up	Temporarily delirious, slightly disoriented
Gulko et al. [23]	1	Left MCA	Small left MCA infarcts	mild-to-moderate right arm and leg extensor weakness	No residual deficits

In our case, the patient showed little improvement in neurologic function throughout admission due to the extensive infarcts and was discharged home on home health services, with tracheostomy and percutaneous endoscopic gastrostomy tube. Further studies in similar cases to identify the cause for differential involvement of the anterior circulation, sparing the posterior circulation, could potentially help with future management in similar critically ill patients.

## Conclusions

COVID-19 infection is associated with stroke due to various proposed mechanisms, including cerebral vasculitis, arterial and venous thrombosis, and hemodynamic hypoperfusion-hypoxic injury, in addition to other CNS involvement such as microhemorrhages, encephalitis, and acute disseminated encephalomyelitis. Vessel wall imaging is a tool that can help differentiate the vasculitis from other mechanisms of stroke in the appropriate clinical context, supported by laboratory evaluations, thereby facilitating appropriate management and preventing potential neurological complications. The differential involvement of the anterior circulation in this case is intriguing, given the extensive involvement, while sparing the posterior circulation. Further studies in similar cases to identify the cause of differential involvement could potentially help the management of critically ill patients due to COVID-19-related cerebral vasculitis.
